# Poster Session I - A36 OPTIMIZING AND STANDARDIZING DONOR SCREENING FOR FECAL MICROBIOTA TRANSPLANTATION: A DELPHI REVIEW

**DOI:** 10.1093/jcag/gwaf042.036

**Published:** 2026-02-13

**Authors:** C S Liu, B Merrick, Z Taboun, B Mullish, E Kuijper, D Kao

**Affiliations:** Division of Gastroenterology, University of Alberta, Edmonton, AB, Canada; Centre for Clinical Infection and Diagnostics Research, King’s College, London, United Kingdom; The University of British Columbia Faculty of Medicine, Vancouver, BC, Canada; Division of Digestive Diseases, Department of Metabolism, Digestion and Reproduction, Faculty of Medicine, Imperial College London, London, United Kingdom; Leiden University Center for Infectious Diseases, Leiden, Netherlands; Division of Gastroenterology, University of Alberta, Edmonton, AB, Canada

## Abstract

**Background:**

Fecal microbiota transplantation (FMT) transfers stool from healthy donors to recipients and is highly effective for preventing recurrent *Clostridioides difficile* infection. However, rare cases of FMT-associated infections have resulted in morbidity and even mortality. Despite the critical importance of screening, evidence-based approaches to developing donor-screening protocols remain limited.

**Aims:**

This review aimed to evaluate the evidence underpinning current FMT donor screening protocols by critically appraising existing literature on pathogen transmission dynamics.

**Methods:**

We conducted a comprehensive review up to November 2024. Key factors considered included the geographical distribution of pathogens, likelihood of fecal-oral transmission via FMT, and the clinical consequences of potential transmission events. A Delphi process involving 25 experts in stool banking, donor screening, and FMT preparation and administration was undertaken to establish consensus on the transmissibility of pathogens via FMT, defined as ≥ 80% agreement. Transmission was categorized as definite when supported by documented evidence (e.g., case reports or confirmed donor-to-recipient transmission), possible when fecal-oral transmission was plausible but unconfirmed, and not possible when such transmission was considered unlikely.

**Results:**

We identified 58 potentially-relevant pathogens and summarized their prevalence, transmissibility, disease severity, and likelihood of FMT-related transmission (Table 1). This was followed by three rounds of the Delphi process generating 21 consensus statements (Table 2), all achieving a consensus of at least 80% except for SARS-CoV-2 (78%). Although fecal-oral transmission of SARS-CoV-2 is unproven, its detection in stool precluded full consensus. Similarly, hepatitis B, hepatitis C, and HIV showed no evidence of FMT-related transmission, though excluding infected donors remains prudent given potential immune and microbiome alterations. Finally, while CMV is typically transmitted via mucocutaneous routes, its transmission was deemed possible based on limited case reports.

**Conclusions:**

This review highlights the need for adopting risk-based screening strategies, refinement of non-infectious exclusion criteria, and consideration of locally-relevant pathogens transmissible via the fecal-oral route. Continued data collection, registry development, and international collaboration are essential to maintain the safety and scalability of FMT in an evolving clinical landscape.

**Funding Agencies:**

None

